# Scheduled simple production method of pseudopregnant female mice for embryo transfer using the luteinizing hormone-releasing hormone agonist

**DOI:** 10.1038/s41598-022-26425-2

**Published:** 2022-12-20

**Authors:** Gema Puspa Sari, Patrick Louis Lagman Hilario, Shunsuke Yuri, Arata Honda, Ayako Isotani

**Affiliations:** 1 Division of Biological Science, Graduate School of Science and Technology, Nara Institute of Science and Technology, 8916-5 Takayama-Cho, Ikoma, Nara 630-0192 Japan; 2 Center for Development of Advanced Medical Technology, School of Medicine, Jichi Medical University, 3311-1 Yakushiji, Shimotsuke-Shi, Tochigi-Ken 329-0498 Japan

**Keywords:** Animal biotechnology, Reproductive biology

## Abstract

The use of mice as experimental animal models has been a practice since the development of genetically engineered mouse models (GEMMs) in the early 1980s. New technologies, including genome editing, have helped in the time- and cost-efficient generation of GEMMs. However, methods for preparing pseudopregnant females, essential for the generation of GEMMs, remain less advanced. This study proposes a new method to achieve simple production of pseudopregnant female mice using a luteinizing hormone-releasing hormone agonist (LHRHa). A 20 µg LHRHa/mouse was identified as the best dose for inducing estrus synchronization. However, the frequency of copulation was 40% on a single injection. With sequential injections of 20 µg LHRHa/mouse on Days-1 and -2, followed by pairing on Day-5, 74% of LHRHa-treated females copulated with male mice. Moreover, LHRHa treatment did not affect fetal and postnatal development. Eventually, successful generation of offspring via embryo transfer was attained using LHRHa-treated pseudopregnant females. LHRHa administration method is efficient in producing pseudopregnant female mice for the generation of GEMMs, and we expect that it will contribute towards advancing the clinical research.

## Introduction

Genetically engineered mouse models (GEMMs), such as knock-in, knock-out, and transgenic mice, have contributed significantly to clinical and biological research, serving as effective disease models. New technologies, including assisted reproductive technology (ART)^1–3^ and gene targeting knock-out technology^4^, have increased the number of GEMMs since the 1980s. Moreover, the advancements in gene-editing technologies, such as CRISPR/Cas9, have helped in the faster, accurate, and efficient development of GEMMs^5,6^.

Since the 1980s, genome engineering and ART have improved in a time- and cost-efficient manner. The ART in mice is on the rise with many recent advancements, including increasing oocyte collection by improving superovulation protocol^7,8^, increased efficiency of in vitro fertilization^9,10^, and improved cryopreservation and resuscitation methods^11–13^. However, the process of embryo transfer has changed little since the 1980s. Producing pseudopregnant female mice as recipients of the embryos in the embryo transfer process remains inefficient.

A pseudopregnant mouse is a female mouse bred with a vasectomized or infertile male mouse to result in sterile mating^14^. Producing pseudopregnant mice requires maintaining approximately 4–5 times the number of females than the actual number that will be used in the embryo transfer process, considering the length of an estrous cycle in mice is usually 4–5 days^15^. Female mice in proestrus and estrus stages need to be selected by visual observation of the vagina before pairing with vasectomized (VAS) males. The accuracy and precision of the female mice selection depend solely on the operator’s training and experience^16,17^. Moreover, since the length of the estrous cycle in mice is 4–5 days (proestrus: < 24 h, estrus: 12–48 h, metestrus; 8–24 h, and diestrus: 48–72 h)^17,18^, operators need to keep more than four times the number of female mice required for the experiment^16,19,20^. Even though methods for producing pseudopregnant female mice have been reported before^15,19,21^, the efficiency of the process depends on the operator’s method, training, and experience, and thus, these methods are relatively tricky for beginner operators.

Several researchers have attempted to overcome these issues by assessing estrus stages by combining visual observation of the vagina and vaginal cytology^19^ and the synchronization of estrus stages^15,22^.

Currently, producing pseudopregnant mice is done by estrus synchronization using progesterone treatment, with 63% of female mice having a vaginal plug after mating^15^. This method is considered the most effective for beginner operators^15^. However, while this method can reduce the number of female mice needed, a high number of male mice is still necessary for the 3 days of continuous pairing with male mice before mating^15^.

In contrast, several efficient methods for producing pseudopregnant female rats using exogenous hormone treatment have been described previously^23–26^.

In this study, we utilized luteinizing hormone-releasing hormone agonist (LHRHa) to synchronize the estrus stage to produce pseudopregnant mice. LHRHa has been known as an agent to regulate fertility since 1971 because of its ability to induce the release of pituitary LH and FSH that drive ovulation in various animals^27^. Moreover, LHRHa can be dissolved in water and administrated through intraperitoneal injection, allowing a simple administration technique for its utilization.

We first determined an effective dose of LHRHa in synchronizing the estrus stage in mice. Following LHRHa-mediated estrous cycle synchronization, production of pseudopregnant mice was scheduled, and we defined the protocol that 74% of female mice were mated with male mice for 1 day only pairing. Finally, we performed embryo transfer with LHRHa-treated pseudopregnant mice as recipients to determine the effects of LHRHa treatment on the reproductive parameters. At last we could establish the new simple method for producing pseudopregnant female mice, which reduce the space and expense in maintaining female stocks and VAS male stocks.

## Results

### Dose optimization of LHRHa for estrus synchronization

The optimal LHRHa dose required for estrous cycle synchronization in mice was identified by administrating different doses of LHRHa in ICR female mice and analyzing their estrus stage using the vaginal cytology method. Borjeson et al.^24^ reported that by administrating 40 μg LHRHa/rat and analyzing the vaginal cytology 96 h later, 55% of female rats were in the late proestrus to estrus stage^24^.

**Figure 1:**
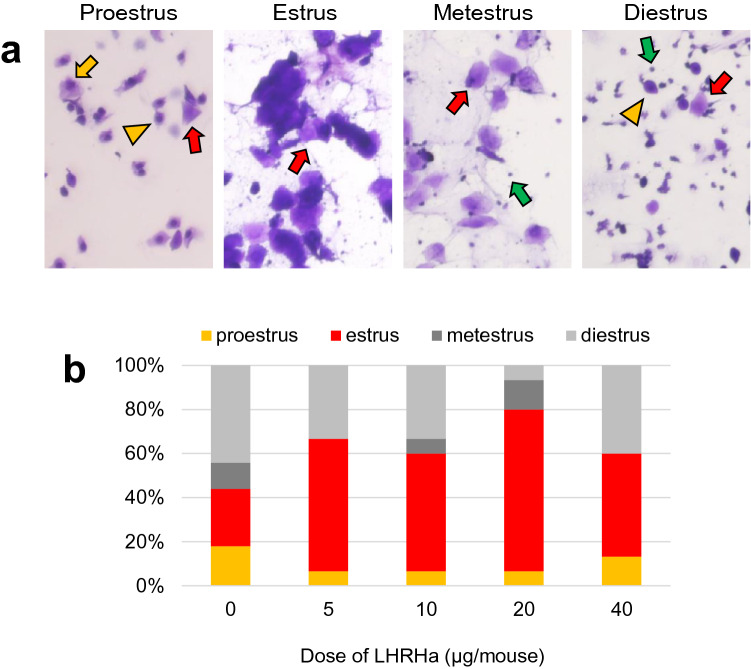
Dose optimization of LHRHa for the estrus synchronization. (**a**) Vaginal cytology of each estrus stage. The proestrus stage includes mainly small nucleated epithelial cells with round shapes, and cornified epithelial cells are also found in late proestrus. The Estrus stage is characterized by predominant anucleated cornified epithelial cells. Metestrus is characterized by a combination of anucleated cornified epithelial cells and leukocytes. Diestrus is characterized by a combination of leukocytes, small and large nucleated epithelial cells, and a low number of cornified cells. The green arrows indicate leukocytes, the yellow arrow indicates large nucleated epithelial cell, the yellow arrow heads indicate small nucleated epithelial cells, and the red arrows indicate anucleate cornified epithelial cells. (**b**) Rates of each estrus stage at 4 days after LHRHa administration in each dose. The actual measurement value was shown in Table 1.

By referring to Borjeson et al.^24^ report, we designed four different doses (0, 5, 10, 20 or 40 μg) of LHRHa. Single dose-LHRHa was intraperitoneally administered to a female mouse, and the vaginal cytology examination was performed 96 h later. The mice who received a dose of 20 μg showed 73.3% (11/15) synchronization in their estrous cycle (Fig. 1 and Table 1), suggesting the estrus synchronization efficacy of LHRHa.

**Table 1:** Estrus stages at Day 4 after LHRHa administration in ICR females.

Dosage	n	Proestrus (%)	Estrus (%)	Metestrus (%)	Diestrus (%)
0 μg^(#1)^	1040	186 (18)	270 (26)	124 (12)	460 (44)
5 μg	15	1 (6.7)	9 (60)	0 (0)	5 (33)
10 μg	15	1 (6.7)	8 (53)	1 (6.7)	5 (33)
20 μg	15	1 (6.7)	11 (73)	2 (13)	1 (6.7)
40 μg	15	2 (13)	7 (47)	0 (0)	6 (40)

^(#1)^Vaginal smear samples were collected from randomly chosen female mice.

### Frequency of copulation after LHRHa treatment

The frequency of copulation after LHRHa treatment was examined based on the presence of vaginal plugs in female mice. On their normal cycle (without any treatment), we confirmed that the frequency of copulation of female mice was 63% (26/41) in proestrus and 64% (114/178) in estrus (Supplementary Table S1). Thus, in the control, the total frequency of copulation during the receptive stage was 64% (140/219).

**Figure 2:**
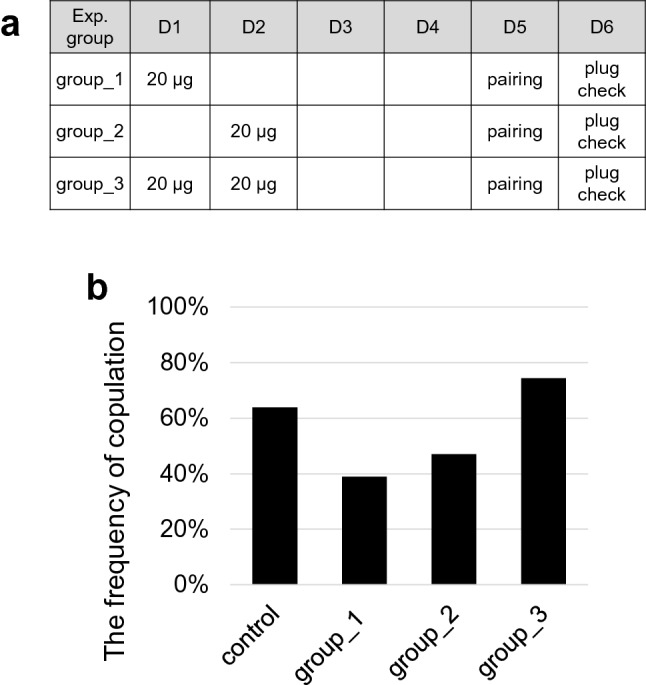
LHRHa dosage conditions and the frequency of copulation. (**a**) Scheme of LHRHa administration schedules and treated LHRHa concentrations. (**b**) Rate of the frequency of copulation with the male after LHRHa administration of female in Fig. 2a conditions. Control means the data using proestrus or estrus stage female mice by vaginal cytology. The actual measurement value was shown in Table 2 and Supplementary Table S1.

The estrus was efficiently synchronized with a dose of 20 μg LHRHa (73.3%, Fig. 1b). Since most of the female mice after LHRH administration were in estrus stage 92–96 h (Day four) later, mating with male mice started at Days four (group_1) or three (group_2) after LHRHa administration (Fig. 2a). Both conditions (group_1: 39%, 14/36; group_2: 47%, 16/34) showed no improvement in the copulation frequency compared with the control (Fig. 2b and Table 2).

**Table 2:** Effects of LHRHa on copulation, and fetal and postnatal development.

Exp. group	No. (%) of females		Litter size^(#3)^	Body weight (g)^(#4)^					
	With plug^(#2)^	Pregnant		P0	n	5w male	n	5w female	n
Control^(#1)^	140/219 (64)	16/16 (100)	13 ± 3	1.9 ± 0.20	192	27.6 ± 4.3	24	24.8 ± 2.0	20
Group_1	14/36 (39)	14/14 (100)	14 ± 4	1.8 ± 0.17 *	187	27.4 ± 2.3	12	24.4 ± 4.0	16
Group_2	16/34 (47)	16/16 (100)	13 ± 5	1.8 ± 0.19 *	199	27.6 ± 1.9	14	25.8 ± 1.9	15
Group_3	29/39 (74)	18/19 (95)	13 ± 4	1.8 ± 0.19	191	26.9 ± 4.5	16	25.7 ± 3.7	18

^(#1)^As a control, the data for calculating the frequency of copulation (data of “with plug”) in Fig. 2 were collected from the proestrus and estrus female mice using vaginal cytology and that in Fig. 3 were collected from LHRHa non-treated plug-positive female mice.

^(#2)^Plug were checked one day after pairing with VAS male or WT mice.

^(#3)^n: All pregnant females in each experimental group.

^(#4)^n: All viable offspring in each experimental group.

Fisher’s exact probability test with Bonferroni correction was performed to calculate the probability of “with plug” and “pregnant” mice. Kruskal–Wallis test with Bonferroni correction was performed for calculating the litter size and body weight of the offspring.

*P < 0.001 vs. the corresponding value of the control.

In a report on the estrus synchronization using progesterone, Hasegawa et al. administered progesterone once a day for 2 days^8^. We attempted a similar condition in group_3 (Fig. 2a). The copulation efficiency in group_3 was improved (74%, 29/39) but had no significant difference compared with the control (Fig. 2b and Table 2). Furthermore, the copulation efficiency was increased in group_3 combined with the visual method (91.6%, 11/12, n = 20). However, four plug-positive female mice were excluded (Supplementary Fig. S2) because they were visually determined to be in non-receptive stages, diestrus or metestrus. Next, we investigated whether the critical point to improve the copulation efficiency was the administration schedule, which is once a day for 2 days or the total dose. Therefore, we attempted five additional conditions described in Supplementary Fig. S1 and Supplementary Table S2. Our results showed that none of these five conditions increased the frequency of copulation compared with the control or the group_3.

### Fetus and postnatal development after LHRHa treatment

The pregnant mare serum gonadotropin (PMSG) has been widely used to induce superovulation in mice and has the ability to synchronize the estrus stage on mice^22,28^. However, PMSG administration can disrupt pregnancy maintenance, affecting the development of the fetus^22,29,30^. In this study, we examined the safety of LHRHa on embryo development.

**Figure 3:**
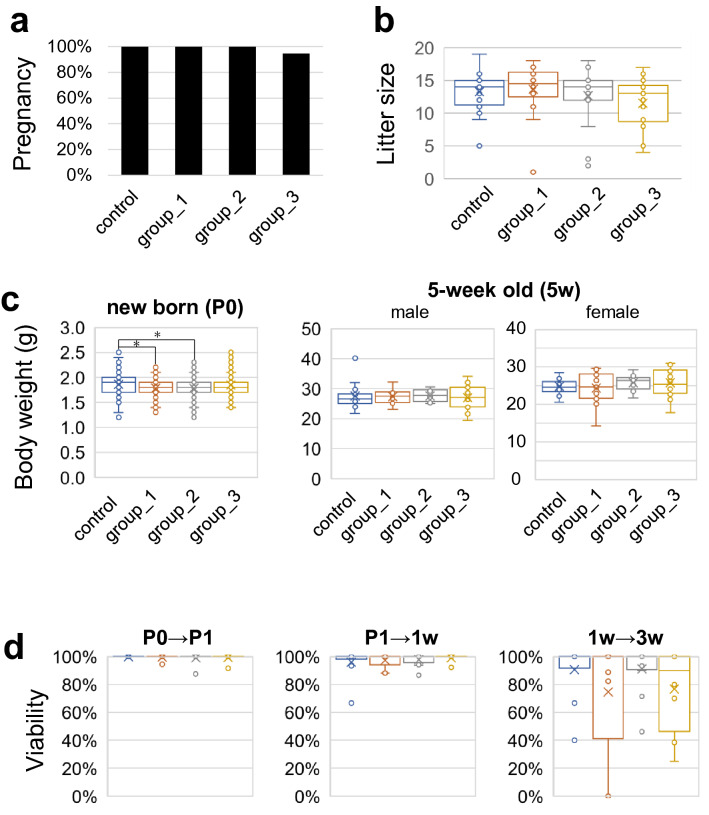
Effects on pregnancy and offspring’s development in the LHRHa-treated female. Data were used for all plug-positive female mice after mating with wild-type ICR male mice in Fig. 2, Table 2, Table 3 and Supplementary Table S3. Control in Fig. 3 means LHRHa non-treated plug positive female mice results. (**a**) Rate of delivered female mice in plug-positive females. (**b**) Litter sizes in each pregnant female mouse group. (**c**) Body weight (g) of offspring from each pregnant female mouse group at E19.5 (P0) and 5-week old. (**d**) Offspring viability 1 day after birth (P0 → P1), from P1 to 1-week old (P1 → 1w), and from 1-week old to 3-week old (1w → 3w). *P < 0.001 vs. the corresponding value of the control. Bonferroni correction on Fisher’s exact probability test was used in a, and Kruskal–Wallis test was used in b, c and d.

We mated the LHRHa-treated female mice with wild-type ICR male mice. The plug-positive female mice were observed and analyzed for their fetal developmental abilities such as pregnancy rate, litter size, the bodyweight, and viability of pups. Except for the body weight of newborn pups, no category showed a significant difference. For the body weight of newborn pups, which means a pup at the date of birth, both group_1 and group_2 exhibited significant differences compared to the control, whereas group_3 did not (Fig. 3c). Therefore, we concluded that none of the categories of LHRHa treatments affected embryo and postnatal development (Fig. 3, Table 2, Table 3 and Supplementary Table S3). Moreover, under group_3 conditions, the body weights of mice that showed no abnormalities in pregnancy and litter development ranged from 27.7 to 37.5 g, as far as we could measure. At least in this range, the LHRHa dosage in group_3 was considered to be effective.

**Table 3:** Effects of LHRHa on offspring viability.

Exp. group	P0 → P1		P1 → 1w		1w → 3w	
	(%) ± SD	n	(%) ± SD	n	(%) ± SD	n
Control	100 ± 0	16	96 ± 10	10	91 ± 20	10
Group_1	100 ± 1	13	97 ± 5	9	75 ± 40	9
Group_2	99 ± 3	15	98 ± 4	12	91 ± 16	12
Group_3	99 ± 2	15	99 ± 3	8	77 ± 28	8

Mean percent viability (%) of all litters from each experimental group and their respective standard deviations (± SD) are shown.

n indicates the number of mouse mothers.

### Generation of pseudopregnant female mice using LHRHa treatment and embryo transfer

After confirming that LHRHa treatment did not affect embryo development, we investigated whether LHRHa treated female mice could be used as pseudopregnant female mice for recipients in embryo transfer. We examined birth rates following embryo transfer in LHRHa-treated group_3 mice. After obtaining pseudopregnant female mice from group_3, we transferred tetraploid complemented embryos into their uterus or in vitro fertilized embryos into their oviduct. Offspring were obtained by natural delivery or Caesarian section on embryonic day 19.5. More than 60% of offspring were survived until weaning (Table 4).

**Table 4:** Embryo transfer using the LHRHa treated pseudopregnant female.

Exp. group	Experiment^(#2)^	ESC lines	Sperm origin	No. of			
				Pseudopregnant	Transferred	Offspring (%)	Wean (%)
control^(#1)^	4n ← ESC	R01-09		2	40	7 (18)	6 (86)
group_3	4n ← ESC	R01-09	N/A	3^(#3)^	30	7 (23)	5 (71)
		*Ets*^*null (em3/em3)*^	N/A		30	9 (30)	6 (67)
	IVF	N/A	GBGS	4	120	22 (18.3)	21 (95)

^(#1)^Pseudopregnant female mice of control were prepared by the visual method.

^(#2)^4n ← ESC refers to embryos manipulated by tetraploid complementation; IVF refers to embryos obtained by in vitro fertilization.

^(#3)^In all three female mice, ten R01-09 embryos were transferred into the left uteri, and ten Ets2 null (em3/em3) embryos were transferred into the right uteri. R01-09 offspring were recognized by RFP fluorescence signal, and Ets2 null (em3/em3) offspring were recognized by wavy hairs, which is known as Ets2-null mutant phenotype, after 10-day old.

*N/A* not applicable.

## Discussion

Synchronizing the estrous cycle increases the efficiency of the production of pseudopregnant mice. Herein, we ascertained whether LHRHa could synchronize the estrous cycle in mice.

We successfully established a scheduled production method for pregnant and pseudopregnant female mice using LHRHa. This method could increase the efficiency in producing pseudopregnant mice compared to the visual method, which is being widely used. While the visual method is the most non-invasive method for selecting the female in producing pregnant and pseudopregnant mice, this method requires long-time training and vast space for keeping numerous mice, both females’ and VAS male stock. Moreover, this method poses a challenge for beginner researchers and small-scale animal facilities^15,16,19^.

We attempted the vaginal cytology method, which is relatively easy for experimental beginners, to assess the estrous cycles before mating. Approximately 60% of female mice, identified as on the receptive stage (proestrus or estrus) by the vaginal cytology method, successfully mated with the male mice (Supplementary Table S1). Usually, at least 50% of the selected mouse in the estrus stage will mate^20^. The presence of a vaginal plug indicates that mating has occurred and must be checked carefully in the early morning, as they could fall out or no longer be detectable 24 h after mating, depending on the mouse strain^31–33^.

The vaginal cytology method is accepted as the most accurate method to evaluate all stages of the estrous cycle in mice^17^. However, the process is laborious and requires a long training period to be a skillful observer, and often disparities among the observers might occur. A report on the estrus stage judgment following vaginal cytology has been developed by Sano et al. to avoid differences in the accuracy depending on the examiners^34^, but it fails to mention the correlation between the estrus stage and the copulation frequency. Although this method helps in selecting the female mice on their receptive stage, a large number of female stocks still needed to be maintained because only 12–15% of female mice were on their estrus stage, based on their natural 4–5 days estrous cycle^19,35^. Therefore, this method alone is not efficient in producing pregnant or pseudopregnant female mice.

Progesterone treatment in mice was shown to synchronize the estrous cycle and was established as a better method than the visual method^15^, as 63% of female mice had a vaginal plug after mating with the male mice. Not only does this reduce the number of female mice required for the experiment, but this method also succeeded in the embryo-transfer experiment by preparing planned pseudopregnant female mice^15^. However, this method required the female to be paired with a male 3 days before mating for efficiency and had a copulation rate of less than 50% if there was no continuous pairing^15^. Thus, the number of male mice required by this method is still high.

Administration of LHRHa at a dose of 20 µg/mouse synchronized the estrous cycle at the estrus stage in most females (73.3%) on Day 4; however, most females were not mating with males (Fig. 1, Fig. 2, Table 1, and Table 2). Subsequent administration of 20 µg/mouse (group_3) LHRHa for 2 days confirmed 74% of copulation frequency with one-day pairing only. The total treated amount of LHRHa in group_3 was 40 µg/mouse and was the same as the dosage for the rat^24^. However, the copulation frequency with one administration of 40 µg/mouse LHRHa conditions (group_7 and group_8) was lower than that of group_3 (Supplementary Table S2). This indicates that the administration condition of LHRHa in mice is different from that of rats, and in this study, the condition of group_3 was shown to be optimal. Moreover, the frequency of copulation in LHRHa-treated group_3 was higher than the one using the vaginal cytology method (64%) (control in Table 2). Interestingly, the mice in group_3 showed higher copulation frequencies than those in group_1, wherein 73.3% of the mice synchronized at the estrus stage. This percentage represented the highest synchronization rate corresponding to the estrus stage (20 µg/mouse in Table 1).

Reportedly, a mouse is sexually receptive when it is either in the proestrus and estrus stage^36^. Our results are consistent with this previous study as shown in Supplemental Table S1. However, the copulation frequency of the female mice was approximately 60% at both the proestrus and estrus stages. This observation suggested that female mice, whose estrous cycle may be the late proestrus to the early estrus stage, have a higher chance of copulation with male mice. Further, given that the number of female mice at the proestrus stage in group_1 was lower than that of the female mice at the metestrus stage, which is the stage that passed the estrus stage (20 µg/mouse in Table 1), it is probable that some of the female mice in group_1 at estrus stage were in the late estrus stage.

Conversely, in group_3 (15%, 3/20), the number of female mice at the proestrus stage was greater than the number of mice at the metestrus stage (5%, 1/20). Therefore, most of the female mice at the estrus stage (65%, 13/20) in group_3 may have been at the early estrus stage. Thus, the frequency of copulation may have increased to a greater extent relative to group_1.

Based on these observations, it is evident that estrus synchronization via LHRHa administration made mating between female and male mice within 1 day possible. Thus, via this strategy, the total number of mice required for mating decreases and scheduled pregnancy as well as pseudopregnancy in female mice becomes possible.

Reportedly, the induction of pseudopregnancy and pregnancy is related to the number and rate of intromission^37^. Additionally, neural stimuli from the vagina, which integrate with other sensory inputs on mating, are essential for initiating the neural and endocrine mechanisms that support pseudopregnancy and/or pregnancy^37^. Therefore, it is necessary to control the estrous cycle as well as the induction of copulation to bring about pseudopregnancy and pregnancy.

Reports have shown that LHRH, also known as gonadotropin-releasing hormone (GnRH), regulates ovulation and promotes female sex behavior, such as lordosis^38,39^. However, progesterone, released from the corpus luteum in the ovary, affects the hypothalamus, including GnRH neurons, and inhibits GnRH secretion. Even though GnRH is indirectly involved in progesterone secretion, the direct function of GnRH is to secrete luteinizing hormone (LH) and follicle-stimulating hormone (FSH) from the pituitary. FSH induces the secretion of progesterone. Therefore, the administration of LHRHa may have effectively synchronized the estrous cycle and induced female sex behavior by producing an LH surge that triggered ovulation. In the absence of mating, the female mice are expected to have their next estrus 4 days later, similar to rats^24^. Synchronization of estrus by utilizing the LHRHa treatment has been reported in big mammals such as sows^40,41^ and gilts^41,42^ for insemination programs, and in small mammals such as rats^24^. Furthermore, by using the LHRHa treatment, the conception rate of females is higher compared to other hormonal treatments such as hCG, as reported in sows^40,41^ and gilts^41,42^.

Both PMSG and LHRHa are similar in their ability to induce estrus synchronization. Even though PMSG treatment shows a high frequency of copulation, our study showed that PMSG-treated mice delivered newborns with low viability. Thus, PMSG is not an excellent option for producing pseudopregnant female mice. Previous studies have also reported harmful effects of PMSG on the fetus development^22,29,30^. In contrast, more than 95% of LHRHa-treated plug-positive female mice became pregnant, and no affected fetal and prenatal development was observed compared with the control (Fig. 3, Table 2, Table 3 and Supplementary Table S3).

While both PMSG and LHRHa can induce estrus synchronization, our study showed they have different effects on fetal development. PMSG has primarily FSH-like and LH-like activity in non-equids^43,44^.

A comparative investigation of these components, such as chemical states, physical properties, and physiological functions, might lead to the development of new strategies for treating miscarriages and newborn mortality in the future.

Overall, we succeeded in establishing an effective production method for pregnancy and pseudopregnancy female mice using LHRHa administration. This method is easy to apply, even for beginner experimenters, and could be performed in small-scale animal facilities. We believe that this study could make the generation of GEMMs more feasible and help progress the research in reproduction and animal biology while supporting the principle of 3Rs- reduction, refinement, and replacement.

## Methods

### Animals

All animal experiments were conducted in accordance with the guidelines of “Regulations and By-Laws of Animal Experimentation at the Nara Institute for Science and Technology,” and were approved by the Animal experimental Committee at the Nara Institute of Science and Technology (the approval no.1639 and 2103). Study of the animal experiments were carried out in compliance with the ARRIVE guidelines^45^**.** ICR mice and 129X1 mice were purchased from SLC (Japan). C57BL/6 N female mice were purchased from Clea (Japan). C57BL/6-Tg(CAG/Acr-EGFP)C3-N01-FJ002Osb (GBGS mouse) was kindly provided by Dr. Ikawa^46^.

The mouse is maintained under 12 h light/ 12 h dark cycle (light on 08:00 AM). Female ages are 8–24 weeks. Male vasectomized mice are prepared by cutting their vas deferens. Food and water are available ad libitum.

### Treatment of the luteinizing hormone-releasing hormone agonist

LHRHa ([des-Gly10, D-Ala6]-LH-RH ethylamide acetate salt hydrate Sigma-Aldrich, L4513) were dissolved in saline. Four conditions of LHRHa (5 μg, 10 μg, 20 μg, 40 μg/mouse) were administrated to 9–24 weeks ICR female mice between 11:00 am and 12:00 am, intraperitoneally. For the vaginal cytology method, LHRHa treated female mice were analyzed 4 days later. For the assessment of the frequency of copulation, and the production of pregnant or pseudopregnant female mice, LHRHa treated female mice were mated with wild type ICR male mice or vasectomized ICR male mice in various conditions, described as Fig. 2a. As the control, non-treated female mice were used in each experiment.

### Cytological assessment of the estrus stage

Cytological examination of the estrus stage was performed as described by Byerset al.^17^ with modification on cell transfer process to the glass slide. Vaginal cells are collected by swab method using a small size cotton-tipped wetted with room temperature phosphate buffer saline (PBS) and inserted into the vagina of the restrained mouse. The cotton tip then turned and rolled carefully against the wall of vagina. The cotton tip was then dipped into 25 ul PBS. The vaginal cells then transferred into glass slide (Matsunami micro slide glass S2112), and then dried inside 56 °C oven for 20 min and then stained with crystal violet (Merck 109218) for 90 s. The slides washed carefully with tap water to rinse the excess staining. After dry, microscopic observation was done under light microscope with 20 × magnification to observe. All cell types samples were collected at 08:00–11:00 am.

### Inducing pregnancy and pseudopregnancy

9- to 24- week-old ICR female mice were paired with wild type ICR male mice for producing pregnant female mice or vasectomized ICR males for producing pseudopregnant female mice. ICR males are housed one per cage with a female introduced at 15:00–16:00 h and vaginal plug was checked at 09:00–11:00 pm the following morning. Females are independently examined for the presence of vaginal plug. The day of plug detection is defined as 0.5-day post coitum (E0.5).

### ES cell culture

R01-09 ESC line was established from embryos by crossing 129X1 female mouse and EGR-R01 ESC (EGR-R01 (CAG-mtDsRed2) BDF1 × 129 Sv) derived male mouse. EGR-R01 ESC line was kindly gifted by Dr. Ikawa. The *Ets2* ^*em3/em3*^ ESC line was reported previously^47^. Establishment of ESC line and maintaining of ESCs were performed as same as previous report^47^. Both ESC lines were provided for tetraploid complementation.

### Tetraploid complementation

Tetraploid embryos were prepared as described previously^47,48^. In brief, ICR two-cell stage embryos were placed in the fusion buffer, and electrofusion was performed by applying 140 V for 50 ms after aligning embryos between the electrodes. CFB16-HB and LF501PT1-10 electrode (BEXCo.Ltd., Tokyo, Japan) were used for cell fusion. Fused embryos were incubated until use. Six to twelve ESCs were injected into a tetraploid four-cell embryo, and then cultured until the blastocysts stage and transferred into the uterus of E2.5 pseudopregnant ICR mice. Offspring were recovered by natural delivery or Caesarean section on E19.5.

### In vitro fertilization

Cauda epididymal spermatozoa from a GBGS male mouse (over 1-year-old) were pre-incubated for 30 min in 100 μl CARD FERTIUP Mouse Sperm Preincubation Medium (Kyudo). Unfertilized eggs with cumulus cells were collected from oviductal ampulla of superovulated C57BL/6 N female mice (8-week-old) in 100 μl HTF medium (ARK Resource) as the insemination drop. Pre-incubated spermatozoa were adjusted to 1 × 10^5^ sperm/ml and incubated with the eggs in the insemination drop at 37 °C under 5% CO_2_ for six hours. Fertilized eggs were washed and move to mWM medium (ARK Resource) and incubated at 37 °C under 5% CO_2_ until the next day. Two-cell stage embryos were transferred into oviducts of E0.5 pseudopregnant ICR mice. Offspring were recovered by natural delivery or Caesarean section on E19.5.

### Assessment of viability

Viability was calculated as the rate of survived pups within a specified time in each litter. In P0 → P1, the number of newborn pups (P0) and pups 24 h after birth (P1) were counted, and percent viability was calculated by dividing the number of P1 pups by that of P0 and multiplying by 100%. This method was applied for calculating percent viability of 1-week old pups per P1 pups, and 3-week old pups per 1-week old pups, indicated as P1 → 1w and 1w → 3w, respectively. Mean percent viability was calculated by averaging percent viability of each litter within an experimental group. Standard deviation for each group was calculated based on their respective mean percent viability.

### Statistical analysis

The statistical difference was determined using Bonferroni correction on the Fisher’s exact probability test for the frequency of copulation and pregnancy rate comparing with the control. Bonferroni correction on Kruskal–Wallis test was used for the litter size, the body weight and viability of pups comparing with the control. A probability of P < 0.01 was considered statistically significant. The statistical difference was calculated using EZR software^49^.

## Supplementary Information

Supplementary Information.

## Data Availability

All data generated or analyzed during this study are included in this published article (and its Supplementary Information files).
