# Supplementary material for: Scheduled simple production method of pseudopregnant female mice for embryo transfer using the luteinizing hormone-releasing hormone agonist
Source: Sci Rep. 2022 Dec 20;12:21985. doi: 10.1038/s41598-022-26425-2 (PMC9767918; doi:10.1038/s41598-022-26425-2)
Supplement: Supplementary file 1 — Supplementary Information. [file 41598_2022_26425_MOESM1_ESM.pdf]

Supplementary information

Scheduled simple production method of pseudopregnant female mice for embryo transfer using the luteinizing hormone-releasing hormone agonist

Gema Puspa Sari [1], Patrick Louis Lagman Hilario [1], Shunsuke Yuri [1], Arata Honda [2], and Ayako Isotani [1*]

1 Division of Biological Science, Graduate School of Science and Technology, Nara Institute of Science and Technology, 8916-5 Takayama-cho, Ikoma, Nara, 630-0192, Japan

2 Center for Development of Advanced Medical Technology, School of Medicine, Jichi Medical University, 3311-1 Yakushiji, Shimotsuke-shi, Tochigi-ken, 329-0498, Japan

*Corresponding author:

Ayako Isotani
E-mail address isotani@bs.naist.jp

Supplementary Fig. S1

Exp. group	D1	D2	D3	D4	D5	D6
group_4	10 µg	10 µg			pairing	plug check
group_5	20 µg	10 µg			pairing	plug check
group_6	10 µg	20 µg			pairing	plug check
group_7	40 µg				pairing	plug check
group_8		40 µg			pairing	plug check

Supplementary Fig. S1

Scheme of LHRHa administration schedules and treated LHRHa concentrations.

Gema PS et al., Supplementary Fig. S2

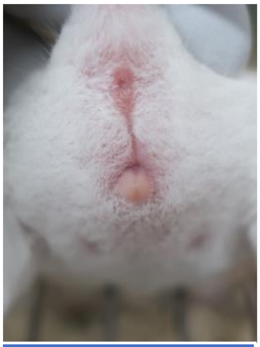

group_3-01	
Visual	-
Plug	-

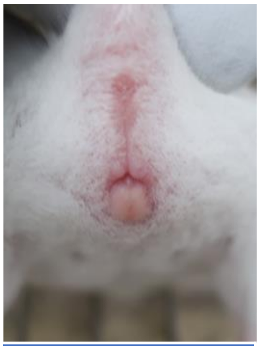

group_3-02	
Visual	+
Plug	+

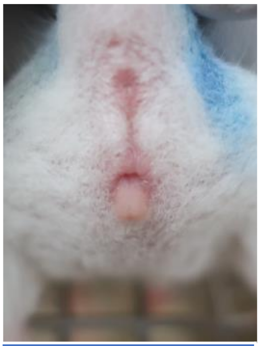

group_3-03	
Visual	+
Plug	+

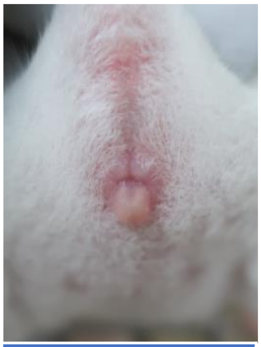

group_3-04	
Visual	+
Plug	-

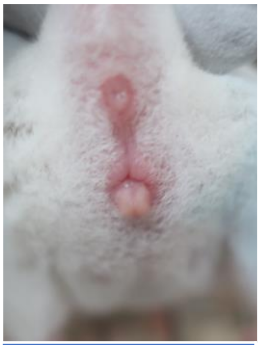

group_3-05	
Visual	+
Plug	+

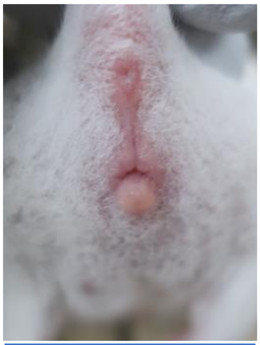

group_3-06	
Visual	+
Plug	+

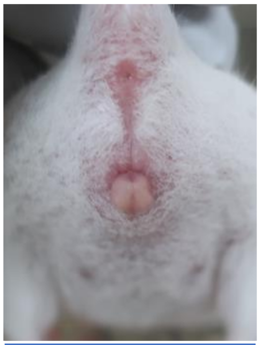

group_3-07	
Visual	-
Plug	-

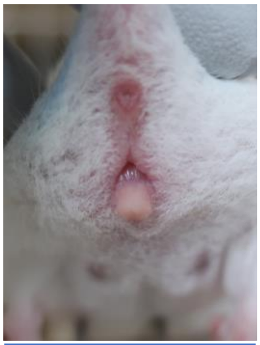

group_3-08	
Visual	+
Plug	+

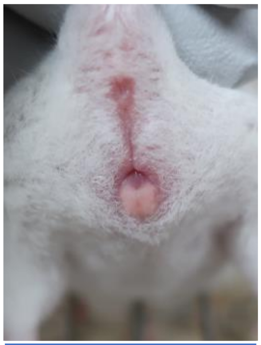

group_3-09	
Visual	+
Plug	+

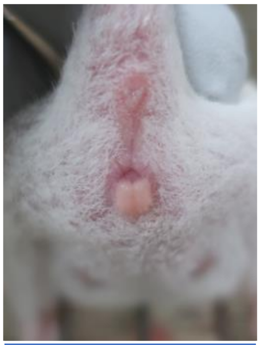

group_3-10	
Visual	+
Plug	+

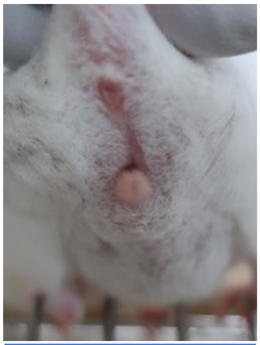

group_3-11	
Visual	-
Plug	-

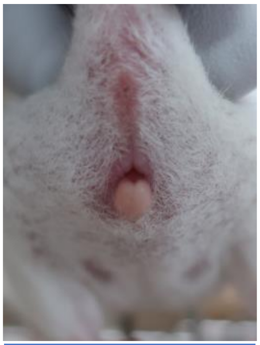

group_3-12	
Visual	+
Plug	+

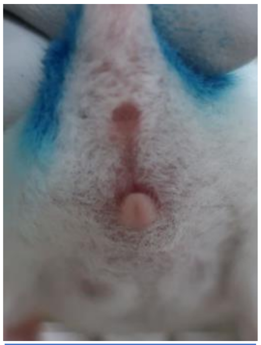

group_3-13	
Visual	-
Plug	+

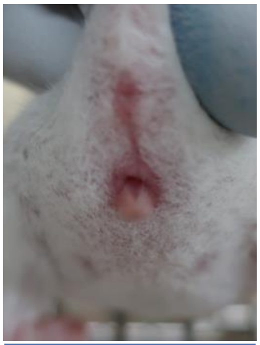

group_3-14	
Visual	+
Plug	+

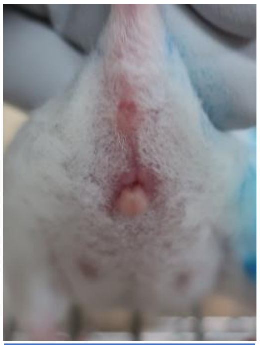

group_3-15	
Visual	+
Plug	+

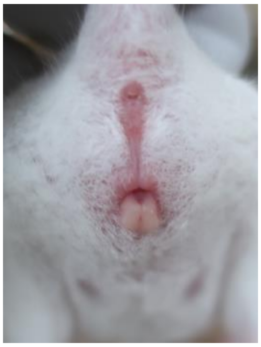

group_3-16	
Visual	+
Plug	+

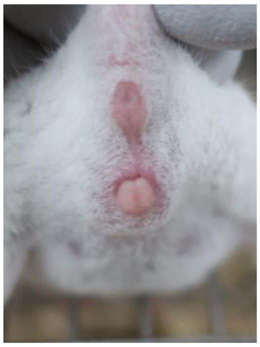

group_3-17	
Visual	+
Plug	+

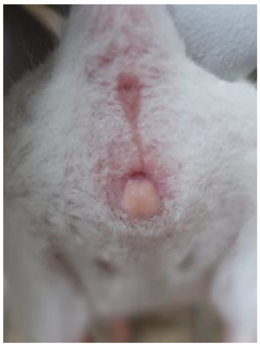

group_3-18	
Visual	-
Plug	+

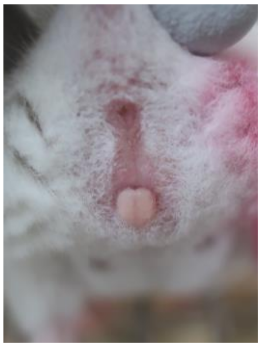

group_3-19	
Visual	-
Plug	+

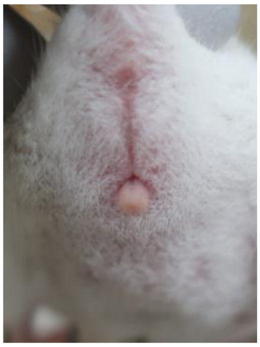

group_3-20	
Visual	-
Plug	-

Supplementary Fig. S2 Pictures of mouse vaginas for visual method after the LHRHa administration in the group_3.
Vaginal pictures were taken just before mating with male mice (D5). Each female mouse was administrated LHRHa in the group_3 condition, judged the potency of mating by visual method, and observed a plug the next day (D6). Visual “+” means the female was judged the high potency of mating with a male. Plug “+” means the female had a plug after mating with a male.

Supplementary Table S1

The frequency of copulation on each estrus stage

Estrus stage	total	plug-positive	%
Proestrus	41	26	63
Estrus	178	114	64
Metaestrus	28	2	7
Diestrus	157	21	13

Supplementary Table S2

Effects of LHRHa treatment by several conditions

exp. group	No. (%) of females		Litter size (#3)	Viability of offspring (%)	Body weight of offspring (g) (#4)
	With plug (#2)	Pregnant			
group_4	6/13 (46)	3/3 (100)	13 ± 1	37/39 (95)	1.9 ± 0.1*
group_5	3/8 (38)	2/2 (100)	14 ± 1	27/27 (100)	1.8 ± 0.2
group_6	4/8 (50)	2/2 (100)	22 ± 1	43/43 (100)	1.8 ± 0.2
group_7 (#1)	1/5 (20)	0/1 (0)	N/D	N/D	N/D
group_8	8/13 (62)	7/7 (100)	15 ± 2	103/103 (100)	1.8 ± 0.1

(#1) Since a plug-positive female mouse in group_7 did not become pregnant, data of offspring were shown as not determined (indicated as N/D).
(#2) Plug were checked one day after pairing with VAS male or WT mice.
(#3) n: All pregnant females in each experimental group.
(#4) n: All viable offspring in each experimental group.
Fisher's exact probability test was performed for With plug, Pregnant and Viability of offspring, and Bonferroni correction on Kruskal-Wallis test was performed for Litter size and Body weight of offspring. * $P < 0.01$ vs. the corresponding value of the control in Table 2.

Supplementary Table S3

Offspring viability of each litter

	exp. group	1	2	3	4	5	6	7	8	9	10	11	12	13	14	15	16	average	SD
P0→P1 (%)	control	100%	100%	100%	100%	100%	100%	100%	100%	100%	100%	100%	100%	100%	100%	100%	100%	100%	0%
	group_1	100%	100%	100%	100%	100%	100%	100%	100%	100%	100%	100%	100%	94%				100%	1%
	group_2	100%	100%	100%	100%	100%	100%	100%	100%	100%	100%	100%	100%	100%	100%	88%		99%	3%
	group_3	100%	100%	100%	100%	100%	100%	100%	100%	100%	100%	100%	100%	100%	100%	92%		99%	2%
P1→1w (%)	control	100%	100%	100%	100%	100%	100%	100%	100%	93%	67%							96%	10%
	group_1	100%	100%	100%	100%	100%	100%	100%	88%	88%								97%	5%
	group_2	100%	100%	100%	100%	100%	100%	100%	100%	100%	94%	94%	87%					98%	4%
	group_3	100%	100%	100%	100%	100%	100%	100%	92%									99%	3%
1w→3w (%)	control	100%	100%	100%	100%	100%	100%	100%	100%	67%	40%							91%	20%
	group_1	100%	100%	100%	100%	100%	89%	82%	0%	0%								75%	40%
	group_2	100%	100%	100%	100%	100%	100%	100%	93%	93%	90%	71%	46%					91%	16%
	group_3	100%	100%	100%	100%	80%	70%	38%	25%									77%	28%
